# Characteristics of the Dye-Sensitized Solar Cells Using TiO_2_ Nanotubes Treated with TiCl_4_

**DOI:** 10.3390/ma7053522

**Published:** 2014-05-05

**Authors:** Jun Hyuk Yang, Chung Wung Bark, Kyung Hwan Kim, Hyung Wook Choi

**Affiliations:** Department of Electrical Engineering, Gachon University, Seongnam-Si, Gyeonggi-Do 461-701, Korea; E-Mails: hyuk1349@gmail.com (J.H.Y.); bark@gachon.ac.kr (C.W.B.); khkim@gachon.ac.kr (K.H.K.)

**Keywords:** TiO_2_ nanoparticle, anodic oxidation, TiO_2_ nanotube, TiCl_4_, dye-sensitized solar cells

## Abstract

The replacement of oxide semiconducting TiO_2_ nano particles with one dimensional TiO_2_ nanotubes (TNTs) has been used for improving the electron transport in the dye-sensitized solar cells (DSSCs). Although use of one dimensional structure provides the enhanced photoelectrical performance, it tends to reduce the adsorption of dye on the TiO_2_ surface due to decrease of surface area. To overcome this problem, we investigate the effects of TiCl_4_ treatment on DSSCs which were constructed with composite films made of TiO_2_ nanoparticles and TNTs. To find optimum condition of TNTs concentration in TiO_2_ composites film, series of DSSCs with different TNTs concentration were made. In this optimum condition (DSSCs with 10 wt% of TNT), the effects of post treatment are compared for different TiCl_4_ concentrations. The results show that the DSSCs using a TiCl_4_ (90 mM) post treatment shows a maximum conversion efficiency of 7.83% due to effective electron transport and enhanced adsorption of dye on TiO_2_ surface.

## 1. Introduction

Since their invention in 1991, dye-sensitized solar cells (DSSCs) have been extensively studied as an alternative to silicon-based solar cells, owing to their simple structure, transparency, flexibility, low production cost, and wide range of application. Despite these advantages, the low efficiency of DSSCs compared to that of silicon-based cells has limited their commercial implementation [1–4]. Consequently, there is a critical need to improve the efficiency of state-of-the-art DSSCs in order to realize next generation solar cells.

DSSCs are composed of four parts as follows: (1) the electrode film layer (TiO_2_), covered by a monolayer of dye molecules, that absorbs solar energy; (2) the conductive transparent conductive oxide layer that facilitates charge transfer from the electrode layer; (3) the counter electrode layer made of Pt or C; (4) the redox electrolyte layer for reducing the level of energy supplied from the dye molecules [5,6]. Thus, research efforts to improve the efficiency of DSSCs have primarily been focused on improvements of the each DSSC component [7]. However, due to synergetic effects of its subcomponents, the enhancement of only one component might not be sufficient to improve efficiency of entire cell.

The interconnected TiO_2_ nanoparticle is widely used as the mesoporous electrode film layer, because it is beneficial for adsorption large amount of dye molecules due to its large surface areas. However, the overall performance of DSSCs can be limited by the electron transport in the nanocrystal boundaries of TiO_2_ nanoparticles and the electron recombination with the electrolyte during the electron migration process. To avoid this problem, many researchers have reported that one dimensional nanostructures can be used in DSSCs as replacements of nanoparticles to facilitate electron transfer [8–14]. In addition to their unique electron properties, one-dimensional TiO_2_ nanostructures also function as light scattering materials. Nevertheless, dye adsorption in the one dimensional structure should be sacrificed due to the reduction of surface area.

In this work, we have considered combined strategies to improve the efficiency of DSSCs. We used oxide semiconductors in the form of TiO_2_ nanotube (TNTs) to improve the electron transport through the film. Though a higher photoelectrical performance was obtained, we believe that further improvements in the photoelectrical performance of DSSCs could be achieved. To overcome reduced dye adsorption in one dimensional structure, we investigated the effects of TiCl_4_ post treatment on DSSCs, combined with variations in the concentration of TNTs in TiO_2_ nanoparticle/TNT composites. Consequently, this approach can be used for effectively increasing the dye adsorption of TiO_2_ films.

## 2. Experimental Section

**Figure 1.**
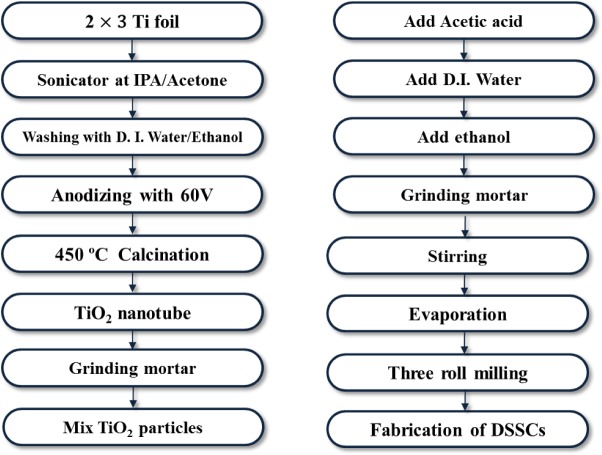
Flow chart of manufacturing dye-sensitized solar cells (DSSCs).

TNTs were prepared by an optimized three step anodization process. Ti foil (0.25 mm thickness, 99.7% purity, Sigma-Aldrich, St. Louis, MO, USA) with an area of 2 cm × 3 cm was degreased by ultrasonic agitation in acetone, isopropanol, and deionized water for 15 min each and then dried with N_2_ gas. The ethylene glycol electrolyte contained 0.25 wt% NH_4_F (98%, Sigma-Aldrich, St. Louis, MO, USA) and 2 vol% deionized water. The anodization was performed in a two electrode system where the Ti foil served as the working electrode and a Pt plate as the counter electrode. Anodization was conducted at room temperature at a constant voltage of 60 V, as shown in Figure 1. In order to obtain powders, the fabricated TNTs had to be detached from the Ti sheet in a H_2_O_2_ solution. The anodic oxidation was repeated many times to obtain the required amount of TNT powder. To achieve TNT powder of the desired crystallinity, the powder was calcined in air at 450 °C for 3 h then the samples were milled with a mortar and pestle. Following this, the TiO_2_ nanoparticles (Anatase 99.9%, Sigma-Aldrich, St. Louis, MO, USA), and the TNT powder were mixed in various ratios (5–20 wt%) and ground in a mortar.

In addition, TiO_2_ paste was prepared by combining TiO_2_ nanoparticles with TNT powder. The prepared TiO_2_ paste was coated onto FTO-glass (Fluorine-doped tin oxide coating glass) by a doctor blade. The TiO_2_ coated substrate was calcined at 250 °C for 15 min and then at 450 °C for 15 min to promote crystal growth and remove organic constituents.

TiO_2_ films were dipped for 30 min in a 30–120 mM TiCl_4_ aqueous solution at 70 °C which was prepared by adding titanium tetrachloride (Sigma-Aldrich, St. Louis, MO, USA) to precooled distilled water in an ice bath. Following the post treatment, the TiO_2_ film was annealed at 450 °C for 15 min.

A Pt catalyst electrode was prepared by mixing H_2_PtCl_6_ (5 mM, Sigma-Aldrich, St. Louis, MO, USA) in isopropyl alcohol with an ultrasonic treatment. A counter electrode, which facilitates the redox reaction of the electrolyte, was fabricated by spin coating the H_2_PtCl_6_ solution at 1000 rpm for 30 s, and annealed at 450 °C for 30 min.

The dye solution to be adsorbed on the electrode films was prepared by mixing 0.5 mM Ru-dye (N719, Solaronix, Rue de I`Ouriette, Aubonne, Switzerland) with ethanol. To facilitate the adsorption of the dye molecules, the prepared TiO_2_ electrode films were placed in the dye solution in darkness for 24 h.

Finally, the DSSC was fabricated by sandwiching the prepared electrode film and counter electrode at 120 °C for 10 min using a hot melt sealant (60 °C). The electrolyte (I^−^/I_3_^−^) was injected between the two electrodes with the inlet then sealed by a cover glass.

The phase of the TNTs obtained by anodization was examined by X-ray diffraction (XRD), using a Rigaku D/max-2200 diffractometer (Rigaku, Tokyo, Japan) with a CuKα radiation source. The morphology of the prepared TiO_2_ films was investigated by field-emission scanning electron microscopy (FE-SEM, Hitachi S-4700, Tokyo, Japan) and the optical transmittance of the prepared TiO_2_ electrode films was measured using a UV-Vis spectrometer (Perkin Elmer Lambda 750, Waltham, MA, USA). The conversion efficiency and electrochemical impedance spectroscopy (EIS, Mcscience K3400, Suwonn-si, Gyeonggi-do, Korea) of the fabricated DSSCs were measured using an *I*–*V* solar simulator (McScience K3000, Suwonn-si, Gyeonggi-do, Korea). The active area of the resulting cell exposed to light was approximately 0.25 cm^2^ (0.5 cm × 0.5 cm).

## 3. Results and Discussion

**Figure 2.**
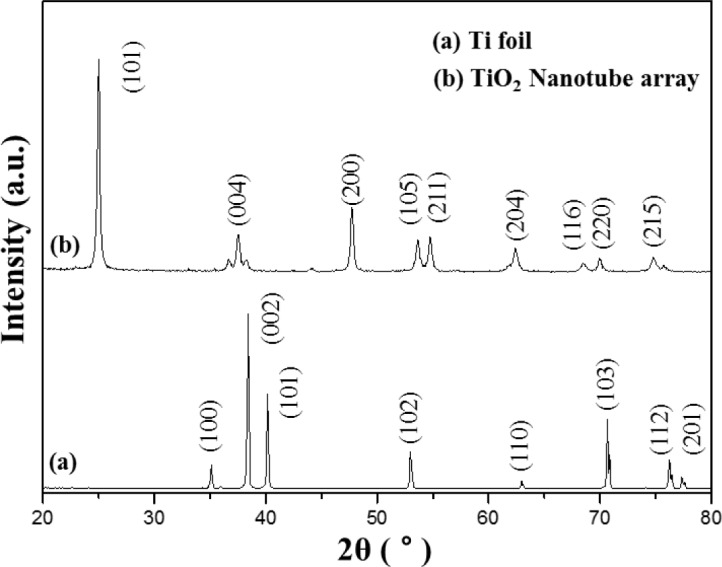
XRD patterns of (**a**) Ti foil and (**b**) a TiO_2_ nanotube array.

Figure 2 shows the XRD pattern of the Ti foil (JCPDS No. 44-1294) and of the TNT array by calcination at 450 °C. After anodization, the TNT array peeled off from the Ti substrate and was analyzed and tested by XRD. The diffraction peaks of TNT array are in good agreement with the standard JCPDS cards of anatase TiO_2_ (No. 21-1272). The XRD pattern of the TNT array shows (101), (004), (200), (105), (211), (204), (116), (220) and (215) anatase peaks.

**Figure 3.**
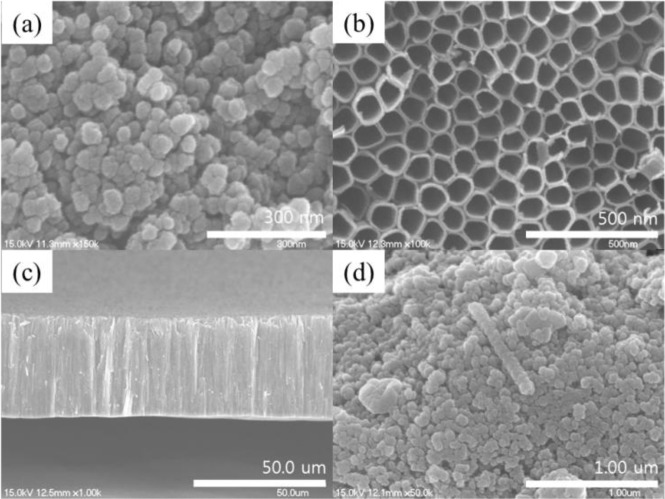
Field-emission scanning electron microscopy (FE-SEM) images of (**a**) TiO_2_ nanoparticles; (**b**) the surface of a TiO_2_ nanotube array; (**c**) a section of a TiO_2_ nanotube array; (**d**) and a TiO_2_ nanoparticle/TiO_2_ nanotube (TNT) composite film.

Figure 3a shows the SEM images of TiO_2_ nanoparticles. The TiO_2_ particle size is about 20–30 nm. Figure 3b indicates that the TNT diameter is about 120 nm, and the TNT surface is uniform. Additionally, for anodic oxidation at present conditions, the TNTs can come to a length of 40–45 μm as shown in Figure 3c. It is obvious that the TiO_2_ nanoparticles, TNTs, and the substrate are well linked, which is helpful for the quick electron transportation in the film. Figure 3d shows the length of TNTs in a TiO_2_ nanoparticle/TNT mixture to be approximately 1 μm.

**Figure 4.**
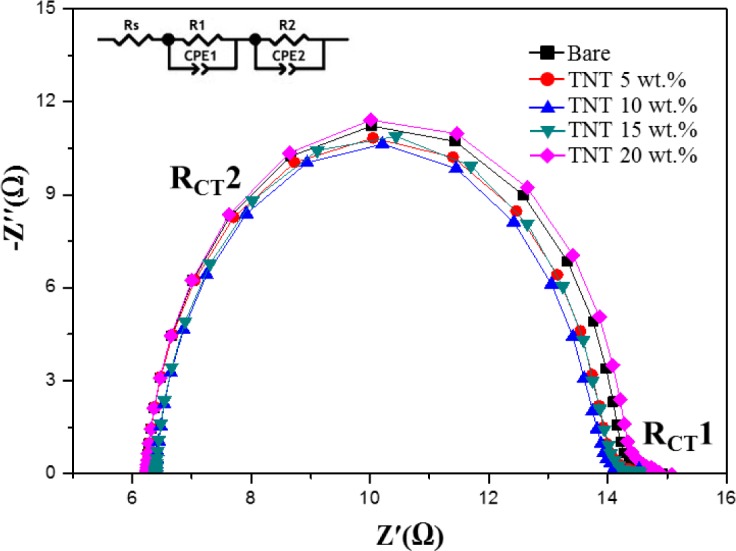
Electrochemical impedance spectroscopy (EIS) Nyquist plots of DSSCs with TNT mixed TiO_2_ films of different TNT concentrations. The following abbreviated terms were used: *R*s (ohmic series resistance), *R*_CT_1, (3 charge-transfer resistance of the counter electrode), CPE1 (constant phase element of the counter electrode), *R*_CT_2 (4 charge-transfer resistance of the working electrode), CPE2 (constant phase element of the photoelectrode).

Figure 4 shows the electrochemical impedance spectroscopy (EIS) analysis of TiO_2_ nanoparticles/TNTs obtained at various weight ratios, which provides information about the electron transport and recombination in DSSCs. Two typical semicircles are observed in Nyquist plots, the small semicircular in the high frequency ranges and the large semicircular in the low frequency ranges correspond to the resistances of Pt/electrolyte interface and electrolyte/dye/TiO_2_ interface. The small semicircle is fit to a charge-transfer resistance (*R*_CT_1) and constant phase, while the large semicircle is fit to a transfer resistance (*R*_CT_2) and constant phase. As *R*_CT_1 is not affected by the use of TiO_2_ nanoparticles/TNTs, we focused on the variations in *R*_CT_2. The first semicircle is a minimum for the TNTs (10 wt%), which is related to charge-transfer resistance of the FTO/TiO_2_ and TiO_2_/electrolyte interfaces (*R*_CT_2). The observed decrease in *R*_CT_2 of TNTs (10 wt%) indicates a reduction in electron recombination and enhancement in the efficiency of electron transport. However, in the case of the TNTs (15 wt%), *R*_CT_2 increased with increasing of TNTs (15 wt%), due to the increase of trap sites which obstructs the movement of electrons from the TiO_2_ film to the photoelectrode [15–18].

**Figure 5.**
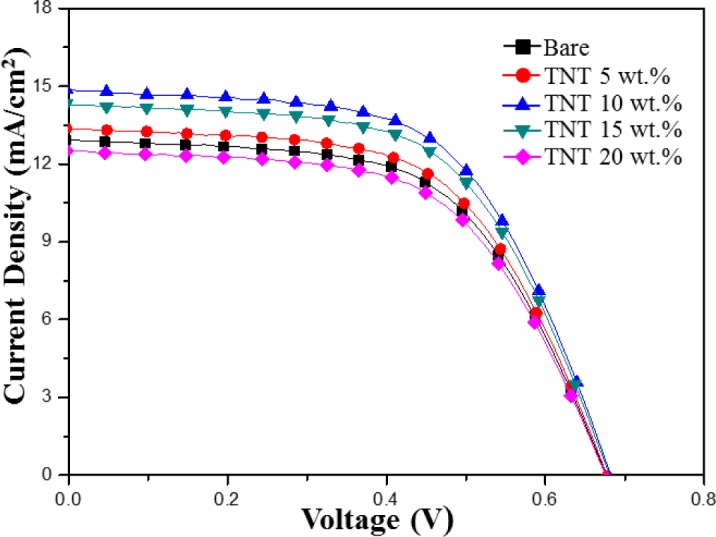
*I*–*V* characteristic of TiO_2_ nanoparticle/TNT DSSCs.

Figure 5 shows the current-voltage photovoltaic performance of DSSCs composed of bare TiO_2_ nanoparticles and TNTs (5–20 wt%) under AM 1.5 illumination (100 mW/cm^2^). Table 1 summarizes the efficiency, fill factor, open circuit voltage, and integral photocurrent for the corresponding solar cells. DSSC with 10 wt% of composite TiO_2_ nanoparticles/TNTs film exhibited the highest light-to-electric energy conversion efficiency of 5.95%, short-circuit current density of 14.86 mA/cm^2^, open-circuit voltage of 0.68 V, and fill factor of 58.79%. These results indicate that the *J*_SC_ value increased significantly with the addition of the TNTs. However, the addition of TNTs had little influence on the open circuit voltage (*V*_OC_) and the fill factor (FF). The observed increase in *J*_SC_ could be attributed to the increased electron lifetime in the one-dimensional electrode on the composite TiO_2_ nanoparticles/TNTs film.

DSSC with TNT (10 wt%) were referred as “Bare” condition (*i.e*., internal reference) in the following measurements to investigate effect of TiCl4 post treatment on DSSCs.

**Figure 6.**
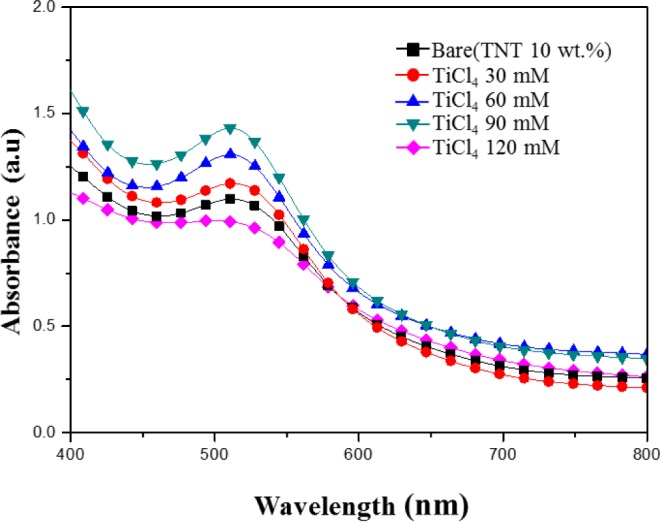
UV-Vis absorbance of TiCl_4_ post treated TiO_2_ films for different TiCl_4_ concentrations.

Figure 6 shows the absorption spectrum of N-719 dye in the 400–800 nm wavelength range in the various TiCl_4_ post treatment (30–120 mM) TiO_2_ films. At the wavelength 400–500 nm, the sample treated with a TiCl_4_ concentration of 90 mM has the highest absorbance. It is reasonable that the TiCl_4_ post treatment electrode provides more sites for dye absorption than the Bare (TNT 10 wt%), leading to a higher light harvesting and *J*sc as expected.

According to Lambert-Beer’s law, higher absorbance means a higher dye concentration. A suitable amount of TiCl_4_ in the film could provide a large surface area for dye adsorption. It is reported that small TiO_2_ particles are formed on the surface of TiO_2_ films by TiCl_4_ post treatment and the surface area and the amount of dye adsorption are increased [19–23]. It is well known that the photocurrent of DSSCs is correlated directly with the number of dye molecules. Therefore, the increase of adsorbed dye molecules results in the increase of incident light being harvested and consequently a larger photocurrent.

In case of TiCl_4_ post treatment with high concentration (120 mM), the absorbance was decrease. The post treatment with the high concentrations can lead the decrease of dye absorption of TiO_2_ film due to the reduction of the film porosity by an increase of the nucleation in the nanoparticles. So, the inefficient charge-transfer paths increase the recombination rate of electrons, as a result, the photocurrent density and conversion efficiency can be decreased [24,25].

**Figure 7.**
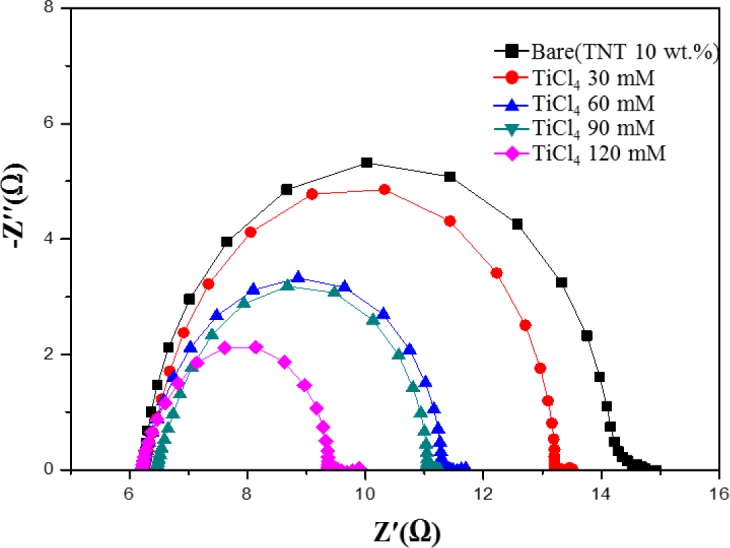
EIS Nyquist plots of DSSCs with TiCl_4_ post treated TiO_2_ films for different TiCl_4_ concentrations.

Figure 7 shows electrochemical impedance spectroscopy (EIS) Nyquist plots of DSSCs with a TiCl_4_ post treatment. EIS is a useful method for the analysis of charge-transport processes and internal resistances [26]. As shown in Figure 7, there is a decrease in the charge-transfer resistance (*R*_CT_) upon increasing the TiCl_4_ ratio from 30 mM to 120 mM. This increases the number of injected electrons into the TiO_2_ film, improves the electrical conductivity, and reduces the charge recombination at the TiO_2_/dye/electrolyte interface [27,28]. *R*_CT_ becomes smaller when the TiCl_4_ ratio increases. The reduction of *R*_CT_ means there is a decrease in the recombination rate and indicates fast electron-transfer processes in the DSSCs. The efficient charge-transfer paths decrease the recombination rate of electrons with I_3_^−^ or the oxidizing dye, resulting in a high photocurrent density and conversion efficiency [24].

**Figure 8.**
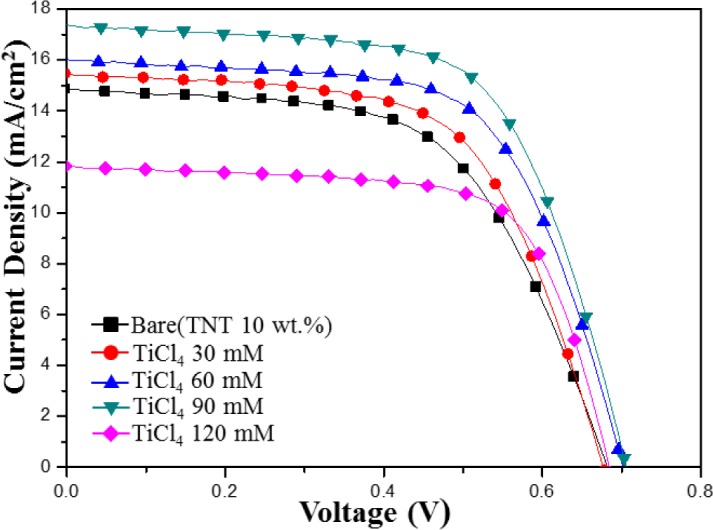
*I*–*V* characteristic of TiCl_4_ post treated DSSCs for different TiCl_4_ concentrations.

Figure 8 shows the *I*–*V* characteristics of the TiO_2_ film with the various TiCl_4_-concentration post treatments. Two of the most important parameters for a solar cell are its photoelectric conversion efficiency and the fill factor (FF). When the *I*–*V* curves approach a square shape, the FF is higher. In addition, solar cells with a high FF have a stable output voltage and current compared to the cell with the same *V*_OC_ and *J*_SC_, and they produce more power. The photovoltaic properties of all post treated films are summarized in Table 2. *J*_SC_ increases with the amount of TiCl_4_ until the TiCl_4_ concentration is 90 mM, beyond this limitation, *J*_SC_ decreases. *J*_SC_ increase was improved due to the increase of dye adsorption and it could be explained by the enhanced loading of dye molecules on TiO_2_ films, which resulted in the improvement of *J*_SC_, and a decrease in the charge-transfer resistance at interfaces. In the case of the TiCl_4_ (120 mM), decrease of *J*_SC_ was result in low absorption of dye from the TiO_2_ film to the photoelectrode.

The FF increased from 58% to 68% after TiCl_4_ post treatment. With optimum post treatment conditions, the DSSCs fabricated on the TiCl_4_ post treatment substrate showed an efficiency value of 7.83% due to an increased photocurrent density and fill factor.

## 4. Conclusions

In this work, the improvement of performance on DSSCs using a TiCl_4_ post-treatment on the TiO_2_ films is proposed. The DSSCs were constructed with TiO_2_ films made from TiO_2_ nanoparticles and TNTs which were fabricated from an anodization process. Without post-treatment, DSSCs with light-to-electric energy conversion efficiency of 5.95% was achieved under a simulated solar light irradiation of 100 mW·cm^2^ (AM 1.5). The DSSCs based on a TiO_2_ nanoparticles/TiO_2_ nanotube composite showed a better photovoltaic performance (higher *J*_SC_) than the cell purely made of TiO_2_ nanoparticles. It was found that the conversion efficiency of DSSCs was highly affected by the properties of TNTs. The effect of a TiCl_4_ post-treatment on the TiO_2_ films was investigated using different the mole ratio of TiCl_4_. DSSCs using TNTs and a TiCl_4_ post treatment were measured to have a maximum conversion efficiency of 7.83% due to effective electron transport. Using TNTs (10 wt%) and a TiCl_4_ (90 mM) post treatment process was found to be an effective method to improve the efficiency of TiO_2_ nanoparticle based DSSCs.

## Figures and Tables

**Table 1.** The integral photocurrent density (*J*_SC_), open circuit voltage (*V*_OC_), fill factor (FF), and efficiency (η) of DSSCs fabricated using pure TiO_2_ particles (bare) and using TiO_2_ particles/TNTs with various compositions.

Sample	V_OC_ (V)	*J*_SC_ (mA/cm^2^)	FF (%)	Efficiency (η%)
Bare	0.67	12.93	58.43	5.11
TNT 5 wt%	0.67	13.37	58.56	5.30
TNT 10 wt%	0.68	14.86	58.79	5.95
TNT 15 wt%	0.68	14.31	58.71	5.71
TNT 20 wt%	0.67	12.52	58.25	4.92

**Table 2.** The integral photocurrent density (*J*_SC_), open circuit voltage (*V*_OC_), fill factor (FF), and efficiency (η) of DSSCs fabricated using TiO_2_ particles/TNTs 10 wt% (bare), and those fabricated using TiCl_4_ post treatment.

Sample	*V*_OC_ (V)	*J*_SC_ (mA/cm^2^)	FF (%)	Efficiency (η%)
Bare (TNT 10 wt%	0.68	14.86	58.79	5.95
TiCl_4_ 30 mM	0.67	15.45	61.37	6.42
TiCl_4_ 60 mM	0.70	16.02	63.75	7.16
TiCl_4_ 90 mM	0.70	17.37	63.84	7.83
TiCl_4_ 120 mM	0.68	11.83	68.53	5.55
